# A community study of the effect of particulate matter on blood measures of inflammation and thrombosis in an elderly population

**DOI:** 10.1186/1476-069X-6-3

**Published:** 2007-02-01

**Authors:** Jeffrey H Sullivan, Rebecca Hubbard, Sally L-J Liu, Kristen Shepherd, Carol A Trenga, Jane Q Koenig, Wayne L Chandler, Joel D Kaufman

**Affiliations:** 1 University of Washington, Department of Environmental and Occupational Health Sciences, Seattle, WA, USA; 2 University of Washington, Department of Biostatistics, Seattle, WA, USA; 3 University of Washington, Department of Laboratory Medicine, Seattle, WA, USA

## Abstract

**Background:**

The mechanism behind the triggering effect of fine particulate matter (PM) air pollution on cardiovascular events remains elusive. We postulated that elevated levels of PM would be associated with increased blood levels of inflammatory and thrombotic markers in elderly individuals. We also hypothesized that elevated PM would increase levels of cytokines in individuals with heart disease.

**Methods:**

We measured these blood markers in 47 elderly individuals with (23) and without (16 COPD and 8 healthy) cardiovascular disease (CVD) on 2 or 3 mornings over a 5 or 10-day period between February 2000 and March 2002. Blood measures were paired with residence level outdoor PM measured by nephelometry. Analyses determined the within-individual effect of 24-hour averaged outdoor PM on blood measures.

**Results:**

Analyses found no statistically significant effect of a same day 10 ug/m^3 ^increase in fine PM on log transformed levels of CRP 1.21 fold-rise [95% CI: 0.86, 1.70], fibrinogen 1.02 fold-rise [95% CI: 0.98, 1.06], or D-dimer 1.02 fold-rise [95% CI: 0.88, 1.17] in individuals with CVD. One-day lagged analyses in the CVD subgroup found similar null results. These same models found no change in these blood markers at the same-day or 1-day lag in the group without CVD. In 21 individuals with CVD, a 10 μg/m^3 ^increase in same-day PM was associated with a 1.3 fold-rise [95% CI: 1.1, 1.7] in the level of monocyte chemoattractant protein-1.

**Conclusion:**

We did not find consistent effects of low ambient levels of PM on blood measures of inflammation or thrombosis in elderly individuals.

## Background

Epidemiological studies have found associations between elevation in levels of fine particulate matter air pollution (PM) and risk of cardiovascular morbidity and mortality in the elderly [1-4]. Moreover, short-term elevations in PM_2.5 _have been associated with triggering the onset of myocardial infarction and AICD discharges [5-7]. However, the mechanism behind this triggering of cardiovascular events by PM_2.5 _remains unclear.

The cardiac effects of air pollution may be induced by a systemic inflammatory response [8-16]. Recent studies indicate that ischemic heart disease is likely mediated at least in part by inflammation [17]. Inflammation plays a substantial role in atherogenic progression, alterations in endothelial function and potentially mediates acute plaque rupture [17,18]. Similarly, *in vitro *and animal studies have highlighted the potential for PM_2.5 _to induce a systemic inflammatory response and systemic oxidative stress that could result in impaired endothelial function [19,20].

Epidemiological studies have suggested that elevated fine PM is associated with increased CRP [21] and fibrinogen [22] levels in healthy middle-age individuals. However, there are inconsistent findings of pollutant effect on fibrinogen after controlled exposures to ambient PM in healthy volunteers [14,23]. Moreover, these controlled exposure studies to concentrated ambient PM have not detected increased levels of interleukin-6 (IL-6), CRP or other pro-atherogenic cytokines [13,14,23]. Last, the only study that assessed the effect of short-term elevations in PM levels on systemic markers of inflammation in a susceptible elderly population found no effect on IL-6 or fibrinogen levels and inconsistent effect on CRP [24]. To determine whether PM_2.5 _is associated with changes in within-individual measures of inflammation or thrombosis, we performed a repeated measures study of these blood markers at the residence level in elderly individuals with and without heart disease. We hypothesized that increased levels of fine PM would be associated with intra-individual increases in CRP levels in those with heart disease. Moreover, we postulated that endothelin-1 and IL-6 levels would be elevated on high air pollution exposure days in individuals with pre-existing heart disease.

## Methods

We performed repeated blood measures of inflammation and thrombosis in 47 elderly individuals with and without cardiovascular disease (CVD) or chronic obstructive pulmonary disease (COPD) participating in a larger prospective study on health effects of air pollution in Seattle [25]. Briefly, subjects were monitored intensively for air pollution exposures and health outcomes over a 10-day period between February 2000 and May 2001, or a 5-day period between December 2001 and March 2002. Blood samples were collected at the subject's home at approximately the same time on two or three mornings during this monitoring period. This blood was assayed for measures of inflammation, and thrombosis. In a subset of 21 participants with CVD, bloods were also assayed for cytokine responses. These data were paired with outdoor nephelometry measures of fine PM made at the individuals' homes. These data were further enhanced by meteorological variables: hourly averages of relative humidity (RH), and temperature; daily questionnaire responses on: medication use (3-hydroxy-3-methylglutaryl coenzyme A reductase inhibitors (statins), aspirin, prednisone, warfarin, heparin, and clopidrogel) and clinic visits. The study protocol was approved by the University of Washington Human Subjects Division.

### Study population

Individuals were eligible if they were living in the greater metropolitan Seattle area and >=55 years of age. The CV population had physician diagnosed ischemic heart disease or congestive heart failure. COPD participants had an FEV1<70% and >40% of predicted. Subjects were excluded if they had unstable angina, myocardial infarction, pneumonia or COPD exacerbation within 30 days of study. Subjects were non-smokers who lived with non-smokers.

### Subject recruitment

Subjects were recruited by advertisement in senior centers and local newspapers and from medical clinics.

### Exposure measures

The primary exposure metric was 24-hour averaged fine PM (from 8-8 am) measured at 0-day and 1-day lags prior to blood measures. PM was measured by nephelometry (Radiance Research, Seattle), as a light scattering coefficient, outside of the participant's residence. Nephelometry data correlate well with gravimetric particle measurements in the 0.1–1.4 aerodynamic range [26]. To allow for comparability to other studies, we calibrated the nephelometric measure of PM against a gravimetric PM_2.5 _measure. In 10 individuals where outdoor nephelometry data were not measured, we used a closest home outdoor nephelometer to capture local PM levels. This nephelometer was located within 2-miles of the subjects' residence. A total of 18 person-days of surrogate nephelometer exposure measures were used in our final analysis.

### Blood measures

Blood samples were drawn from an antecubital vein from the seated subject following the recommendations of the International Committee for Standardization in Haematology[27]. Blood samples were immediately placed on ice and centrifuged within 2 hours. The serum was stored at -70°C for later batched analysis.

High sensitivity C-reactive protein (CRP) was measured using the Dade-Behring radioimmunoassay method[28]. This assay had a lower limit of detection at 0.02 mg/l. The intra-assay coefficient of variation was less than 5%. Fibrinogen levels were measured in citrated plasma by the Clauss method[29]. D-dimer was measured in citrated plasma using an enzyme immunoassay (Diagnostica Stago). D-dimer results are reported in ng/ml of fibrinogen equivalent units (FEU).

The measures of endothelin-1(ET-1), interleukin-6(IL-6), interleukin-6 receptor (IL-6r), tumor necrosis factor-α (TNF-α), tumor necrosis factor-receptors (*p55, p75*) and monocyte chemoattractant protein-1(MCP-1) were performed in duplicate using standard ELISA techniques (R&D Systems, Minneapolis). The intra-assay coefficient of variation for ELISA assays ranged from 2.7% for IL-6 to 5% for ET-1.

### Statistical analysis

The data were analyzed using the statistical package SAS (version 8.02, Cary NC). The inflammatory and thrombotic measures were log transformed prior to analyses. For ease of clinical interpretation, we present the data after the beta coefficient has been converted to a natural scale. Effect sizes can be interpreted as the multiplicative change in mean outcome associated with a 10 ug/m^3 ^increase in fine PM.

#### Primary analysis

We used a mixed model with random intercepts for unique subject-sessions (SAS Proc Mixed procedure) to determine the within-individual effect of zero-day and one-day lagged 24-hour averaged outdoor PM on measures of inflammation (CRP and fibrinogen) and thrombosis (D-dimer) for individuals in each health group. Final models were adjusted for age, gender, medication use, and meteorological variables (temperature and relative humidity as both linear and quadratic terms), and the interaction of outdoor PM with anti-inflammatory medication use (aspirin, prednisone) and statin use were also considered. We found no significant interaction effect of medication use with fine PM and report only overall effects.

#### Secondary analysis

An analysis restricted to those with pre-existing heart disease, used a linear mixed effects model to determine the within subject-session effect of a zero-day or one-day lagged 10 μg/m^3 ^increase in fine PM on the log-transformed levels of MCP-1 and ET-1 after adjusting for age, gender, medication use, temperature and relative humidity (as linear and quadratic terms), and the interaction with medication use.

## Results

We performed 133 plasma measures of inflammation and thrombosis in 47 individuals with (23) and without (16 COPD and 8 Healthy) cardiovascular disease over 23 study sessions. The overall study population was older (median age 77, range 56–89) and 43% were female (Table 1). The CV and non-CV study populations did not differ by demographic factors (Table 1). The CV population was composed of 6 individuals with heart failure and 17 individuals with ischemic heart disease without overt heart failure. Statin drugs were used in 31% of individuals with heart disease.

**Table 1:** Summary of Demographic and Clinical Characteristics of Study Participants.

Variable		Cardiovascular N = 23	COPD N = 16	Healthy N = 8
Age	Median (range)	77 (56, 86)	80 (65, 89)	78 (66, 88)
Gender	Female	10 (43%)	6 (38%)	4 (50%)
Race	White	23 (100%)	16 (100%)	7 (88%)
History of MI	Yes	9 (39%)	3 (19%)	0 (0%)
Diabetes	Current	4 (17%)	1 (16%)	1 (13%)
Angina	Current	6 (26%)	3 (19%)	0 (0%)
CHF	Current	7 (29%)	0 (0%)	0 (0%)

Table 2 demonstrates that the range of exposure decreased when restricting to session and within-individual concentration of outdoor PM_2.5_. Accounting for concentrations measured on 133 subject-days across study sessions, the median concentration of 24-hour averaged outdoor PM_2.5 _was 7.7 μg/m^3 ^(range 1.3–33.9). Restricting exposure to within the 23 study sessions, the within session median 24-hour averaged outdoor PM concentration was 6.4 μg/m^3 ^(range 0.7–23.7 μg/m^3^). Within individual, within session median 24-hour averaged outdoor PM concentration was 4.7 μg/m^3^(range 0.06–23.7 μg/m^3^). Only 6 individuals (4 CV and 2 non-CV) experienced an intra-session variation of 24-hour averaged PM_2.5 _exposure of greater than 15 μg/m^3^.

**Table 2:** Summary of the 24-hour Average Pollutant Exposures Preceding Blood Measure in the Study Participants.

**Variable (unit)**	**N**	**Min**	**25%**	**50%**	**75%**	**90%**	**Max**
^a^Outdoor PM_2.5 _(μg/m^3^)							
Subject-days	133	1. 3	5.2	7.7	11.5	19.9	33.9
Session	23	0.7	4.0	6.4	9.7	17.1	23.7
Subject-session^b^	44	0.06	2.5	4.7	9.4	16.5	23.7
^a^Indoor PM_2.5 _(μg/m^3^)							
Subject-days	107	1.3	5.4	7.7	12.1	16.0	81.4
Central Site (Study-days)							
NO_2 _(ppb)	51	0.01	0.02	0.02	0.03	0.03	0.03
Temperature (F)	58	35.5	41.4	45.3	50.4	57.0	67.8
Relative Humidity	58	55.5	76.0	82.1	87.5	94.2	100.0

^a^Represents PM_2.5 _equivalent of actual nephelometric measure averaged from 8 am on prior day to 8 am on day of study.

^b^Subjects with greater than two 24-hour average measures of PM_2.5 _equivalent during study session.

The 1-day lagged measures are very similar; therefore, they are not displayed.

Table 3 summarizes our measures of inflammation and thrombosis. These data demonstrate similar thrombotic and inflammatory marker levels in the cardiovascular, pulmonary and healthy subgroups. Interestingly, the individuals with cardiac disease have a median value of CRP [0.20 mg/l] that was within the lowest quintile of CRP distribution in apparently healthy elderly participants in other studies (17). Last, the range of values of CRP, D-dimer and fibrinogen in this study population were consistent with prior results in elderly populations (30).

**Table 3:** Summary of Inflammatory and Thrombotic Blood Measures by Health Status

Health Status	D-dimer ng/ml (median: range)	CRP mg/l (median: range)	Fibrinogen mg/dl (median: range)
Healthy [n = 8]	503: 353 – 2020	0.30: 0.10 – 2.0	392: 313 – 511
COPD [n = 16]	427: 163 – 1164	0.60: 0.10 – 3.5	425: 327 – 617
Cardiovascular [n = 23]	368: 162 – 4359	0.20: 0.02 – 14.5	403: 268 – 674

Our analyses found no effect of fine PM at 0-day lag on CRP 1.2 fold-increase [95% CI: 0.9, 1.6], fibrinogen 1.0 fold-increase [95% CI: 1.0, 1.1] or D-dimer 1.1 fold-increase [95% CI: 0.9, 1.2] in those with CV disease after adjusting for RH and temperature. The 1-day lagged analyses in the CV subgroup found similar null results, Table 4. These same models found the same null association between fine PM and these blood measures in the healthy and COPD subgroups, Table 4. Further stratification of these models by covariates known to influence measures of thrombosis including age, gender, anti-inflammatory or anticoagulant medication use, diabetes, active angina or CHF did not modify the null association between fine PM and CRP, fibrinogen and D-dimer levels in the CV, COPD or healthy subgroups, data not shown.

**Table 4:** Fine PM effect on blood markers of inflammation and thrombosis in study participants.^+^

Disease Status	24-hr average PM lag-day	CRP Fold-rise (95% CI)	Fibrinogen Fold-rise (95% CI)	D-dimer Fold-rise (95% CI)
CV	0-day	1.19 (0.86, 1.64)	1.02 (0.97, 1.07)	1.05 (0.91, 1.22)
	1-day	1.25 (0.97, 1.58)	1.01 (0.97, 1.05)	1.04 (0.93, 1.15)
COPD	0-day	0.93 (0.48, 1.79)	1.00 (0.91, 1.08)	1.04 (0.93, 1.17)
	1-day	0.69 (0.34, 1.42)	1.05 (0.97, 1.13)	1.10 (0.95, 1.28)
Healthy	0-day	0.97 (0.48, 1.07)	0.93 (0.88, 1.00)	1.02 (0.80, 1.31)
	1-day	1.01 (0.85, 1.19)	0.88 (0.81, 0.95)	1.10 (0.76, 1.58)

^+^The models control for relative humidity and temperature.

^†^Model assesses affect of a 10 ug/m^3 ^increase in fine PM on blood markers.

Moreover, using these same models, we found no effect from a 1-ppb increase in either 0-day or 1-day lagged 24-hour average NO_2 _or a 1-ppm increase in 0-day or 1-day lagged 24-hour average CO level on inflammatory or thrombotic measures in the CV, COPD or healthy subgroups, data not shown.

Our final model did not find a significant effect of a same day 10 ug/m^3 ^increase in fine PM on measures of CRP 1.21 fold-rise [95% CI: 0.86, 1.70], fibrinogen 1.02 fold-rise [95% CI: 0.98, 1.06], or D-dimer 1.02 fold-rise [95% CI: 0.88, 1.17] in individuals with pre-existing CV disease (Table 5). These same models found no change in these blood markers in the COPD or healthy subgroups, (Table 5). Moreover, stratifying by statin use did not modify the absence of association between 0-day or 1-day lagged increased PM and levels of CRP or fibrinogen in CV, COPD or healthy subgroups, data not shown.

**Table 5:** Change in markers of inflammation and thrombosis in participants from a 10 ug/m^3 ^increase in PM_2.5_.^+^

Disease Status	24-hr average Outdoor PM lag-day	CRP Fold-rise (95% CI)	Fibrinogen Fold-rise (95% CI)	D-dimer Fold-rise (95% CI)
CV	0-day	1.21 (0.86, 1.70)	1.02 (0.98, 1.06)	1.02 (0.88, 1.17)
	1-day	1.25 (0.97, 1.58)	1.0 (0.97, 1.03)	1.03 (0.93, 1.15)
COPD	0-day	0.93 (0.48, 1.80)	1.0 (0.91, 1.09)	1.04 (0.93, 1.16)
	1-day	0.69 (0.33, 1.46)	1.08 (0.99, 1.17)	1.09 (0.94, 1.27)
Healthy	0-day	0.98 (0.88, 1.08)	0.94 (0.87, 1.01)	0.95 (0.79, 1.14)
	1-day	1.01 (0.84, 1.21)	0.99 (0.88*, 1.17)	0.97 (0.71, 1.31)

^+ ^The model is adjusted for age, gender, medication use, temperature, and relative humidity.

We analyzed PM effect on cytokine levels in 21 individuals with known cardiac disease by pairing 59 repeated-measures of endothelin-1, IL-6 and IL-6 receptor, TNF-α and TNF-α receptors [p55, p75], and MCP-1 to outdoor residence level measures of PM. The median level of MCP-1 was 156 pg/ml (range: 66–647 pg/ml) and the median level of endothelin-1 was 3.6 pg/ml (range: 1.5–6.6 pg/ml). We were unable to assess the effect of PM on levels of TNF-α and IL-6 as most values were below the limit of detection of our assays, which were 2.0 pg/ml and 0.3 pg/ml, respectively.

Our analyses found that a 10 μg/m^3 ^increase in zero-day outdoor PM was associated with a 1.3 fold increase [95% CI: 1.1, 1.7] in the level of MCP-1 in these individuals after controlling for age, gender, RH, temperature and medication use (Table 6). This effect was not evident at the 1-day lag exposure 1.0 [95% CI: 0.9, 1.3]. A 10 μg/m^3 ^increase in PM was not associated with a significant increase in ET-1 levels at the 0-day lagged exposure 1.0 [95% CI: 0.8, 1.2] and 1-day lagged exposures 1.1 [95% CI: 0.9, 1.2] (Table 6). The use of statin medications did not modify the association between the same day fine PM levels and levels of MCP-1 or ET-1.

**Table 6:** Effect of a 10 ug/m^3 ^increase in PM_2.5 _on cytokine levels of participants with cardiovascular disease.^+^

Lag-day	MCP-1 Fold-rise (95% CI)	Endothelin-1 Fold-rise (95% CI)
0-day	1.3 (1.1, 1.7)	1.1 (0.8, 1.2)
1-day	1.0 (0.9, 1.3)	1.1 (0.9, 1.2)

^+ ^Models were adjusted for age, gender, medication use, temperature, and relative humidity.

* The majority of measures of TNF-alpha and IL-6 were below the limit of detection of assays; therefore, we were unable to determine effect of PM on these markers.

## Discussion

Our study was unable to find consistent associations between increased PM_2.5 _levels and elevations in blood indices of inflammation or thrombosis. Our finding of an increased MCP-1 level from a 10 ug/m^3 ^increase in fine PM should be interpreted with caution given the multiple analyses conducted in this project.

Although *in vitro *and animal studies support an inflammatory mechanism of PM effect [31-34], it is possible that PM exerts cardiac effect through direct interaction with the heart and vascular endothelium [35] or through neurogenic mechanisms [15,36]. We chose our blood measures to reflect the production of acute-phase proteins (CRP and fibrinogen) and circulating inflammatory mediators (cytokines) that characterize the systemic inflammatory response. Therefore, our null results likely reflect the small variation in PM_2.5 _exposure over the study sessions; inadequate control for personal and medication effects on blood measures; and within-subject physiologic variability of these blood markers.

Small excursions in fine PM levels may not result in appreciable changes in blood measures of inflammation or thrombosis even in susceptible individuals. Our null findings for IL-6, CRP and fibrinogen are consistent with a repeated blood measures study in 108 elderly individuals performed over an 18-month interval by Seaton that only found consistent increases in red blood cell indices (packed cell volume) from the multiple blood measures of inflammation and thrombosis [24]. Our results are also in agreement with controlled exposure studies to ambient PM in healthy volunteers that find no effect of PM on multiple blood markers of inflammation and thrombosis with the exception of fibrinogen. Our results contrast with results from a cohort study of 631 middle-age men in Germany' that found a greater than 2-fold risk of CRP level>99% (10.8 mg/l) from a 5-day average 26 ug/m^3 ^increase in total suspended particles (TSP)[21]. Due to differences in exposure measures (3-day and 5-day moving averages) and analytic method in this study, it is difficult to make a direct comparison of relative effect of PM between studies. Our results are consistent with a PM effect on CRP between a 20 percent decline to a 70 percent increase in blood measure.

The absence of PM effect on blood markers of inflammation and thrombosis at exposure levels of 50–120 ug/m^3 ^PM_2.5 _in both healthy and asthmatic young volunteers suggests that an inflammatory effect of fine PM may be limited to susceptible populations [14]. Despite their underlying medical conditions, our subjects did not generally have elevated levels of inflammatory measures over the study. This may suggests that our small sample of elderly individuals are not those at greatest risk of PM effect. Epidemiological studies suggest that acute bronchitis, and pneumonia potentiate the cardiac effect of PM in individuals with underlying cardiovascular and pulmonary disease [37]. The mechanism behind the augmentation of PM effect is unclear, but may be explained by changes in transcription of pro-inflammatory genes secondary to the acute infection. Moreover, we speculate that the null results could reflect differences in genetic determinants of susceptibility to PM. Individuals with GSTM1 null and GSTP1 1/1 genotypes have demonstrated increased sensitivity to diesel particulate matter and environmental tobacco smoke suggesting that common genetic variants may influence susceptibility to PM [38]. Unfortunately, our small sample size does not permit further gene-environment analyses as a source of the null results.

The inherent physiologic variability of our inflammatory and thrombotic measures may outweigh the small effects from PM even after restricting our change in blood measure to a within-person, within-session effect. We may have inadequately controlled for disease-related (silent ischemia, CHF and COPD exacerbations, and medication use) and non-disease related factors (stress, depression) that are known to transiently increase the levels of inflammatory and thrombotic markers independently of PM effect.

Last, the higher fraction of secondary aerosols rich in sulfates and transition metals composing PM in the European studies may account for the difference in effect of PM on inflammation and thrombosis between the studies.

Our study had several strengths that add to the validity of results. It was performed in an elderly population with and without cardiac and respiratory disease. Our intensive residential level exposure monitoring potentially decreased exposure misclassification. We controlled for diurnal variation in inflammatory and thrombotic markers by performing blood measures at the same time in each individual.

## Conclusion

Our small study was unable to find a pro-inflammatory or pro-thrombotic effect of fine PM at low ambient levels. As the mechanism of PM effect on cardiovascular function remains unknown, we encourage performance of systemic measures of inflammation, thrombosis and oxidative stress in larger panel studies of susceptible populations and in controlled exposure studies to ambient PM in elderly individuals with and without cardiac and respiratory disease.

## Competing interests

The author(s) declare that they have no competing interests.

## Authors' contributions

JS contributed to the study design, acquisition of data, analysis and interpretation of data and drafting and revision of manuscript. RH and KS contributed to statistical analyses, interpretation of data and revisions of manuscript. CT contributed to study design, interpretation of data and critical revisions of manuscript. SL contributed to the study design, exposure assessment, drafting of the manuscript and critical revisions of manuscript. JQK contributed to study concept, study design, data acquisition and manuscript revision, WC contributed to study design, measurement of blood markers, interpretation of results and manuscript revision. JDK contributed to study design, data interpretation, manuscript preparation and critical revisions of manuscript.
